# Tinnitus and Reactions to Tinnitus: A Cross-Sectional Survey Across Different Tinnitus Durations

**DOI:** 10.3390/audiolres16030064

**Published:** 2026-04-27

**Authors:** Anna Carolina Marques Perrella de Barros, Joel Isaac Berger, Richard S. Tyler

**Affiliations:** 1 Obermann Center of Advanced Studies, University of Iowa, Iowa City, IA 52245, USA; annacaro-marquesperrelladebarros@uiowa.edu; 2 Tinnitus and Sound Intolerance Group, Universidade Federal de São Paulo, São Paulo 04021-001, Brazil; 3 Department of Neurosurgery, University of Iowa, Iowa City, IA 52245, USA; joel-berger@uiowa.edu; 4 Department of Otolaryngology, University of Iowa, Iowa City, IA 52245, USA; 5 Department of Communication Sciences and Disorders, University of Iowa, Iowa City, IA 52245, USA

**Keywords:** tinnitus, surveys and questionnaires, time factors, demographics

## Abstract

**Background/Objectives:** Tinnitus and reactions to the tinnitus are different dimensions that can be explored in research and in clinical settings. Notably, these dimensions can elucidate priorities and the most problematic areas for patient-centered approaches. The aim of this study is to determine how tinnitus is perceived and impacts people who have experienced tinnitus for different durations. **Methods:** People with tinnitus were invited to participate in a survey at the University of Iowa Tinnitus Website. 709 people responded and documented their perceived sound, problems experienced, and duration of tinnitus. We assessed correlations between the duration of tinnitus and the pitch rating, the loudness rating, and the Tinnitus Primary Function Questionnaire scores. Additionally, we performed a multiple linear regression analysis, considering the dependent variable ‘duration of tinnitus’, to explore associations between duration of tinnitus and the aforementioned factors. This was a cross-sectional study based on comparisons of responses from patients with different tinnitus durations, rather than examining the same patients longitudinally. **Results:** The analysis demonstrated that respondents with a longer duration of tinnitus reported higher loudness ratings (*p* = 0.010). However, their reactions to tinnitus (Tinnitus Primary Function Questionnaire) were associated with a decrease compared with a shorter duration of tinnitus (*p* = 0.048). There was no association between pitch rating and duration of tinnitus. **Conclusions:** Our findings indicated louder tinnitus was associated with a longer duration of tinnitus. However, in general, the functional impact of the tinnitus was associated with a decrease. Notably, there was considerable variability among individuals, suggesting that additional factors contribute to these relationships. These findings can be considered in treatment decisions and counseling strategies.

## 1. Introduction

Tinnitus—the perception of an auditory stimulus in the absence of an external source—has many different causes and mechanisms [1,2]. To date, there are no universally effective treatments for tinnitus, which may in part be explained by differential reactions that people have to experiencing tinnitus. A psychological model developed by our group distinguishes tinnitus from the reactions to the tinnitus [3]. There are beneficial counseling and sound therapy approaches to help people cope with their tinnitus. In exploring treatments to reduce or eliminate tinnitus, it would be helpful to understand how and why reactions to tinnitus may change across individuals. The aim of the current study was to investigate tinnitus perception (loudness, pitch) and tinnitus reactions (Tinnitus Primary Function Questionnaire) [4] across individuals with different tinnitus durations.

## 2. Materials and Methods

In this cross-sectional study, we performed an online survey. Patients responded to a variety of questions about their tinnitus. The survey was made available through the University of Iowa Health Care Tinnitus and Hyperacusis website. Tinnitus subjects could find the survey through several internet searching approaches.

Participants were questioned about their age, gender, where they perceived their tinnitus, the best quality that described their tinnitus, and how long they have had tinnitus. They were asked to rate tinnitus pitch, loudness, and complete the Tinnitus Primary Function Questionnaire (TPFQ) [4]. Loudness and pitch rating scales were numerical and varied from 0 to 100 for loudness (very faint to very, very loud), and from 1 to 100 for pitch (very low to very high). Numerical rating scales provide a large range for high resolution and are intuitive to patients. We analyzed responses from different patients with varying tinnitus durations.

All participants provided informed consent to use their data. No personal identification information was collected. Study protocols were approved by the University of Iowa Institutional Review Board.

Participants with self-reported tinnitus were recruited over a six-year period. To ensure data quality and prevent duplicate responses, the survey platform was configured to restrict access based on IP address validation, limiting each unique identifier to a single online evaluation. All data parameters and quality control measures remained under the direct supervision of the investigators.

### Data Analysis

Statistical analyses were performed using jamovi (Version 2.6) [5], running on the “R statistical environment” (Version 4.4) [6]. Variables were summarized using means and standard deviations. Distributional characteristics were examined prior to inferential testing. Inferential analyses used the nonparametric Mann–Whitney test when comparing analyzed variables within the subgroups of tinnitus duration.

Correlational analyses were performed to examine the relationship between the duration of tinnitus and the pitch rating, the loudness rating, and the TPFQ, reporting Spearman’s correlation coefficient values (ρ). Statistical significance was defined as *p* < 0.05. Correlation coefficients were interpreted according to conventional effect size criteria [7]: |ρ| = 0.10–0.29 (weak), 0.30–0.49 (moderate), and ≥0.50 (strong).

Multiple linear regression analysis was performed to determine whether the duration of tinnitus (dependent variable) significantly explained variance in pitch rating, loudness rating, and the TPFQ variables. Tinnitus duration was analyzed as a continuous dependent variable to examine between-subject variability in tinnitus chronicity. Regression coefficients (β) and 95% confidence intervals were reported.

## 3. Results

This study involved 709 subjects (325 females and 384 males), aged 18 to 85 years. The age, gender and tinnitus characteristics are presented in Table 1.

The TPFQ (N = 449) showed scores that ranged from 33 to 89.9, with standard deviations considerably high (~35–40) (Table 2), highlighting that there were large inter-individual differences. Lower scores were observed in questions 14 and 17 (tinnitus interfering with speech), 16 (disrupted sleep).

**Figure 1:**
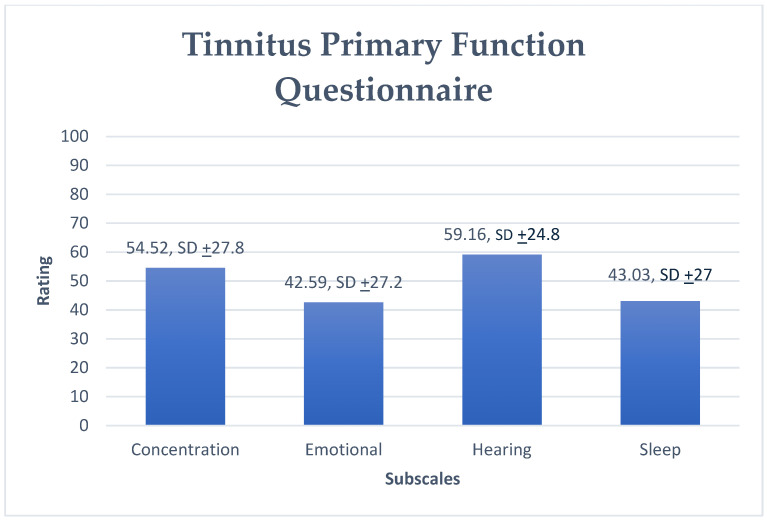
Mean rating scores for the Tinnitus Primary Function Questionnaire’s scales.

The mean scores of TPFQ scales demonstrated greater impact for the Hearing domain (Questions 2, 6, 9, 14; mean 59.16, SD ± 24.8), followed by Concentration (Questions 3, 7, 11, 15, 19; mean 54.52, SD ± 27.8); Sleep (Questions 5, 13, 16, 18, 20; mean 43.03, SD ± 27), and Emotions (Questions 1, 4, 8, 10, 12; mean 42.59, SD ± 27.2), highlighting that these latter domains had a lower impact on the population of the present study (Figure 1).

Comparative analyses were performed on subgroups to report TPFQ scores (N = 449), pitch ratings (N = 709), and loudness ratings (N = 709) across different tinnitus durations. The discrepancy in sample size between the full cohort (N = 709) and the TPFQ subset (N = 449) is due to incomplete responses in the TPFQ section of the survey. As participation was voluntary and the questionnaire was self-administered, some participants did not complete all sections, particularly those presented later in the survey. Missing data were handled using a complete-case approach; thus, only participants with valid TPFQ responses were included in analyses involving this measure.

Comparing people who had tinnitus for 1 year or less with those who had tinnitus for more than 1 year, there were no statistically significant differences regarding TPFQ ratings (*p* = 0.429), pitch rating (*p* = 0.884), or loudness rating (*p* = 0.856). Ratings were also compared between individuals who reported experiencing tinnitus for a maximum of 5 years and those who reported having tinnitus for more than 5 years. There were likewise no differences between these subgroups regarding pitch rating (*p* = 0.533) and loudness rating (*p* = 0.376). A statistically significant difference was observed in the TPFQ ratings analysis (*p* = 0.036) (Table 3). The subgroup with tinnitus for more than 5 years had lower TPFQ scores than the subgroup that presented tinnitus for 5 years or less. Comparing results of those with tinnitus for a maximum of 10 years or more than 10 years, there were no statistically significant differences for the variables analyzed (*p* > 0.05).

Correlation analyses between the duration of tinnitus and pitch rating, loudness rating, and TPFQ are presented in Table 4. There was no association between the duration of tinnitus and the pitch rating (*p* = 0.117). Loudness rating had a significant positive correlation with duration of tinnitus (*p* = 0.010). Loudness ratings were slightly higher in people who reported long-duration tinnitus. The TPFQ and the duration of tinnitus were significantly negatively correlated (*p* = 0.048). This finding revealed that the challenges documented in the TPFQ likely decrease for many with longer tinnitus durations.

These relationships were further investigated while controlling for intercorrelations among predictors. Consequently, we performed a multiple linear regression analysis, considering ‘duration’ as the dependent variable (Table 5). This analysis confirmed that loudness ratings showed a positive association with tinnitus duration (β = 0.060, *p* = 0.043), TPFQ scores were negatively associated with tinnitus duration (β = −0.084, *p* = 0.009), and that pitch ratings were not associated with tinnitus duration (β = 0.024, *p* = 0.405).

**Figure 2:**
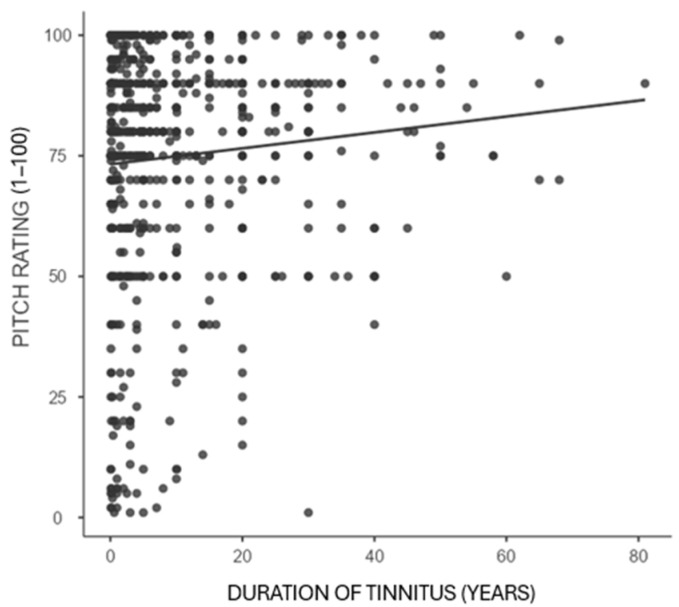
Correlation between pitch rating and duration of tinnitus. Legend: *p*-value = 0.117, correlation = 0.059.

**Figure 3:**
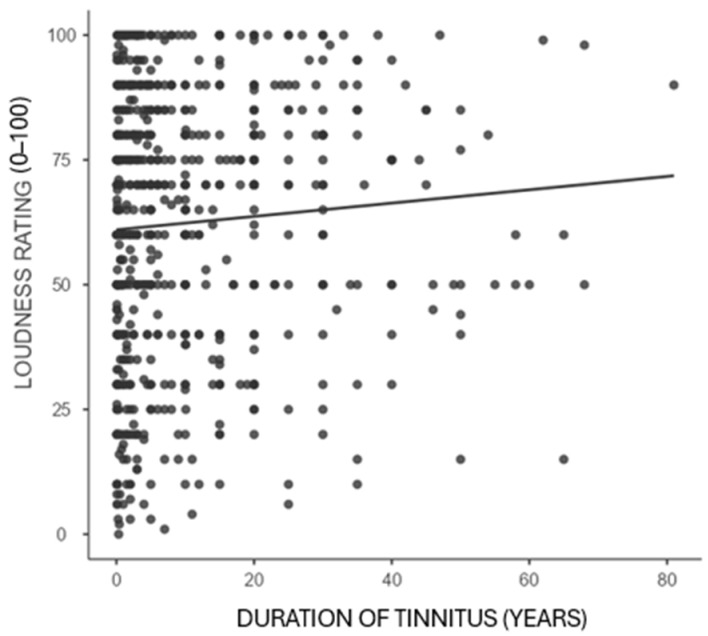
Correlation between loudness rating and duration of tinnitus. Legend: *p*-value = 0.010, correlation = 0.098.

**Figure 4:**
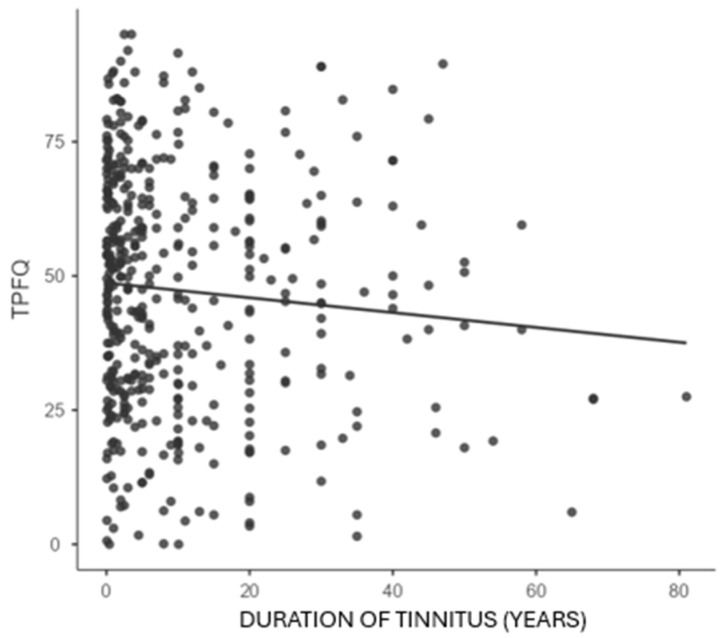
Correlation between TPFQ and duration of tinnitus. Legend: *p*-value = 0.048, correlation = −0.094.

Bivariate correlations are shown in Figure 2, Figure 3 and Figure 4, in order to illustrate relationships between tinnitus duration and pitch rating, tinnitus duration and loudness rating, and tinnitus duration and TPFQ scores, with the line of best fit overlaid on each figure.

Nonparametric (Spearman’s test) and parametric (multiple linear regression) analyses presented a consistent pattern of results. Notably, the associations observed in our study are robust across statistical approaches. It is important to highlight that effect sizes were small (|ρ| ~0.09–0.10), suggesting relatively modest associations between tinnitus duration and the variables analyzed, and highlighting that several other factors contribute to variability across individuals.

## 4. Discussion

People with a variety of tinnitus experiences completed the questionnaire. Only a few reported tinnitus in their head (14.4%). Most presented tinnitus perception in one (23.8%) or both ears (61.2%) (Table 1). Ringing-whistling type was the most common quality of tinnitus (44.6%). Mean scores of tinnitus pitch and loudness rating were high (over 60/100). The average duration of tinnitus was approximately 10 years.

The tinnitus percept itself and the reactions to the tinnitus percept are two separate entities [3]. Tinnitus can certainly affect people in different ways. The reactions people have—focused within the current study on how tinnitus affects Sleep, Hearing, Concentration, and Thoughts & Emotions—also vary dramatically (Table 2). This was explored with the TPFQ [4]. Some individuals get used to their tinnitus after a few months, but most do not. This is important to consider when deciding on the appropriate steps to take in counseling.

The Hearing subscale of the TPFQ was the most impacted by tinnitus according to respondents (Figure 1). Prevalence of hearing loss among tinnitus subjects is high (~90%) [8,9]. Tinnitus itself can cause hearing problems; thus, it is important to distinguish difficulties caused by tinnitus or hearing loss [10]. Although the TPFQ attempts to distinguish hearing difficulties caused by tinnitus from those caused by hearing loss, this can be difficult for respondents to distinguish. Tinnitus can interfere with afferent and efferent auditory pathways [11]. Additionally, tinnitus can lead to worse performance in auditory central processing [12,13,14,15].

The Concentration subscale was also highly affected by the presence of tinnitus (Figure 1). Tinnitus can affect the ability to focus and concentrate [10,16]. Tinnitus subjects are distracted by their tinnitus, and this affects their attention [17,18,19]. Assessing top-down attentional control in subjects with tinnitus can highlight inhibitory control or controlled attention problems [20].

A comparison of results between respondents with tinnitus for a maximum of 5 years and those with tinnitus for more than 5 years revealed that tinnitus distress can be lower in subjects with longer-standing tinnitus (Table 3). However, this is variable across individuals and can vary with the presence of stress.

Problems faced by tinnitus patients also include those related to thoughts and emotions [10,16]. People have concerns and problems that can affect their emotional well-being and lead to increased distress [10,16]. This may be elucidated by questions regarding the impact of tinnitus on their hearing and mental illness and concerns that tinnitus can lead to senility. All of these concerns reinforce the importance of understanding how tinnitus may affect cognition. Tinnitus has emotional valence and becomes the focus of selective attention [21]. These aspects are part of the Tinnitus Activities Treatment, which is a patient-centered counseling approach for tinnitus management, helping patients with education on coping strategies [10,16,22,23].

Frustration can also contribute to distress in the first few years after tinnitus onset. Many with tinnitus hope for a “magic pill” that could totally eliminate tinnitus perception [24,25]. Tinnitus sufferers dedicate several efforts (including financial) to searching for a quick solution. However, this often leads to frustration. This is another factor that highlights the importance of counseling in tinnitus management. There are many paths to treatment, including counseling and sound therapy. The patient should be made aware of helpful options.

The correlation analysis within the current study demonstrated that respondents with a longer duration of experiencing tinnitus reported modestly higher loudness ratings. However, as many respondents experience their tinnitus over a long period of time, some adapt and report lower TPFQ scores (Table 4). There was no significant association between the pitch rating and the duration of tinnitus. Bivariate regressions confirmed these findings (Table 5) and showed the same directions for these associations (Figure 2, Figure 3 and Figure 4).

Slightly higher loudness ratings were associated with long-term tinnitus. This is an important factor, as hearing loss increases over time, resulting in additional challenges. Many conditions lead to a progressive decrease in hearing, such as noise exposure, presbycusis, and possibly hereditary hearing loss [26]. This underscores the need to understand whether the problems experienced with tinnitus might be related to the progression of hearing loss. Therefore, hearing loss affects tinnitus perception, with a similar effect to being in quiet rooms, and can contribute to more intrusive tinnitus [27]. It demonstrates the importance of periodic audiological assessments and the benefits of providing hearing aids to some individuals.

Additionally, it is relevant to emphasize the difference between the subjective perception of the tinnitus loudness (obtained by ratings) and tinnitus by psychoacoustic measures of loudness matching. Clinically, some patients report loudness increase, but psychoacoustic measures remain stable. The salience network related to tinnitus can contribute to an increased loudness. Tinnitus is processed by interacting networks, including brain regions involved in salience detection, memory, and emotional processing [28,29]. The ascending auditory pathway acts as the earliest location in which neural changes co-occur with tinnitus, which may itself influence tinnitus loudness codification [30]. Brain areas responsible for salience processing of tinnitus can also play a role in conscious perception of tinnitus and contribute to loudness perception [28].

Participants with a longer duration of tinnitus reported a lower impact of tinnitus (measured by the TPFQ). This finding may be related to habituation, as an effect of longterm exposure to tinnitus [2]. Counselling and coping strategies also may contribute to a lower tinnitus distress associated with longer tinnitus duration and can facilitate habituation [31]. Emotional stress is a significant indicator of tinnitus severity [32]. It is possible that people with a longer duration of tinnitus can have fewer emotional reactions to the symptom, as some individuals may partially adapt.

Differential effects were observed between subjective perception of loudness and the perception of the functional impact of tinnitus among participants with longer tinnitus duration. This may highlight a partial adaptation to tinnitus, considering the persistent perceptual characteristic of loudness. Additionally, this finding reinforces that psychoacoustic characteristics are not necessarily related to the reactions of tinnitus [3,33]. Some previous studies suggest that tinnitus loudness remains stable [34] or may decrease [35] over time. Our results further suggest that distress tends to decrease and can be indicative of habituation [34,35].

The perceived effects of tinnitus vary significantly across different duration-based cohorts. Effects can vary widely among sufferers. In some individuals, the tinnitus loudness can increase. For others, it can decrease, making it easier to adapt to. Furthermore, for most individuals, hearing loss will increase over time (due to presbycusis). This certainly can impact tinnitus reactions. The effects of aging can impact some people’s ability to cope with life’s challenges, including coping with tinnitus. Attention and emotional state can modulate tinnitus perception. Periods of anxiety in everyday life can influence people’s ability to cope with their tinnitus [28,36,37]. Tinnitus can certainly affect one’s quality of life [37,38,39].

Although the statistical analyses showed a consistent pattern of results, the effect sizes were small. This suggests modest associations between tinnitus duration and the variables analyzed and highlights inter-individual variability that is not fully accounted for within the current study. Additionally, this emphasizes how tinnitus is a heterogeneous condition. Tinnitus and tinnitus reactions can vary substantially. It is important to note that the present study did not actually follow individual patients over time. Instead, we examined responses from different individuals who had tinnitus for different durations. Longitudinal studies are required to understand how reactions can change within individuals. The potential influence of prior or ongoing treatments for tinnitus was not controlled in our study. We also note that the recruitment strategy adopted may result in an overrepresentation of patients with greater symptom burden. Objective audiological data, hearing aid adoption, and knowledge of the underlying etiology of tinnitus would have been beneficial to contribute to a more comprehensive interpretation of the findings.

## 5. Conclusions

Our findings show the wide variation of tinnitus and reactions to tinnitus in different people. People with longer durations of tinnitus reported louder tinnitus. However, it is true that many people adapt to their tinnitus after longer periods of symptoms. A very important point is the wide variation of tinnitus percepts and reactions to tinnitus. Additionally, it is important to understand how the presence of tinnitus can affect each individual’s life. People with tinnitus often present with hearing and communication problems, especially different degrees of hearing loss. For some, these factors result in work obstacles. For others, there are significant obstacles in interacting with partners, family, and friends. These observations have a major impact on counseling, providing sound therapy, medications, and other treatments.

## Figures and Tables

**Table 1:** Study Sample Characterization.

	Study Population (N = 709)	
Age (years)	52.27 (SD ± 14.4)	
	N	%
Gender		
Female	325	45.8
Male	384	54.2
Where is Your Tinnitus		
Head	102	14.4
Outside of head	4	0.6
Ear	603	85.0
Both Ears	434	61.2
One Ear	169	23.8
Quality of Tinnitus		
Buzzing	53	7.5
Cricket-like	69	9.7
Hissing	130	18.3
Humming	28	3.9
Ringing Whistling	316	44.6
Roaring Shhh Rushing	66	9.3
Other	47	6.6
Tinnitus Duration (years)	9.97 (SD ± 13.2)	
Tinnitus Pitch Rating (1–100)	74.58 (SD ± 24.1)	
Tinnitus Loudness Rating (0–100)	63.23 (SD ± 26.3)	

Legend: SD, Standard Deviation.

**Table 2:** Responses to Tinnitus Primary Function Questionnaire; (see Appendix A). (SD, Standard Deviation).

	1	2	3	4	5	6	7	8	9	10
Mean	86.4	44.0	38.7	56.8	48.4	58.3	53.5	41.7	44.1	48.8
Median	100	40	25	70	40	70	60	30	50	50
SD	24.8	39.3	37.0	39.8	39.2	40.3	37.4	38.0	39.0	39.0
	11	12	13	14	15	16	17	18	19	20
Mean	44.1	89.9	44.3	36.9	47.1	36.4	33.0	40.7	54.1	47.4
Median	40	100	30	25	50	20	20	30	50	45
SD	37.0	23.8	39.8	38.8	36.7	35.5	35.5	37.9	37.5	39.5

**Table 3:** TPFQ rating responses before and after 1, 5, and 10 years of tinnitus duration.

		N	Mean	Median	SD	*p*-Value	Effect Size
TPFQ	~1 year	43	49.8	52.8	20.8	0.429	−0.073
	>1 year	406	47.3	48.1	21.9		
	~5 years	172	50.3	52.8	21.3	0.036 *	−0.118
	>5 years	277	45.8	46.5	22.0		
	~10 years	233	48.3	49.8	21.9	0.505	−0.036
	>10 years	216	46.8	47.8	21.8		

Legend: * *p* < 0.05.

**Table 4:** Correlation between the duration of tinnitus and pitch rating, loudness rating, and Tinnitus Primary Function Questionnaire.

		Pitch Rating	Loudness Rating	TPFQ
Duration	Correlation	0.059	0.098	−0.094
	*p*-value	0.117	0.010 *	0.048 *
	CI (95%)	[−0.012; 0.135]	[0.025; 0.171]	[−0.183; 0.000]

Legend: * *p* < 0.05; TPFQ, Tinnitus Primary Function Questionnaire; CI, Confidence Interval.

**Table 5:** Multiple linear regression analysis between the duration of tinnitus (dependent Variable) and pitch rating, loudness rating, and Tinnitus Primary Function Questionnaire.

		Pitch Rating	Loudness Rating	TPFQ
Duration	β	0.024	0.060	−0.084
	*p*-value	0.405	0.043 *	0.009 *
	CI (95%)	[−0.033; 0.081]	[0.001; 0.118]	[−0.146;−0.021]

Legend: * *p* < 0.05; TPFQ, Tinnitus Primary Function Questionnaire; CI, Confidence Interval.

## Data Availability

The datasets presented in this article are not readily available because access is controlled by the Institutional Review Board. Requests to access the datasets should be directed to Richard Tyler.

## Abbreviations

The following abbreviations are used in this manuscript:

TPFQ	Tinnitus Primary Function Questionnaire

## Appendix A

Iowa Tinnitus Primary Function Questionnaire (20-Item Version) [4]

Please indicate your agreement with each statement on a scale from 0 (completely disagree) to 100 (completely agree).

Concentration

3. When there are lots of things happening at once, my tinnitus interferes with my ability to attend to the most important thing.

7. I feel like my tinnitus makes it difficult for me to concentrate on some tasks.

11. I have difficulty focusing my attention on some important tasks because of tinnitus.

15. My inability to think about something undisturbed is one of the worst effects of my tinnitus.

19. I have trouble concentrating while I am reading in a quiet room because of tinnitus.

Emotion

1. My tinnitus is annoying.

4. My emotional peace is one of the worst effects of my tinnitus.

8. I am depressed because of my tinnitus.

10. I am anxious because of my tinnitus.

12. I just wish my tinnitus would go away. It is so frustrating.

Hearing

2. My tinnitus masks some speech sounds.

6. The effects of tinnitus on my hearing are worse than the effects of my hearing loss.

9. My tinnitus, not my hearing loss, interferes with my appreciation of music and songs.

14. In addition to my hearing loss, my tinnitus interferes with my understanding of speech.

17. One of the worst things about my tinnitus is its effect on my speech understanding, over and above any effect of my hearing loss.

Sleep

5. I have difficulty getting to sleep at night because of my tinnitus.

13. The difficulty I have sleeping is one of the worst effects of my tinnitus.

16. I am tired during the day because my tinnitus has disrupted my sleep.

18. I lie awake at night because of my tinnitus.

20. When I wake up in the night, my tinnitus makes it difficult to get back to sleep.
